# A Single-Source Dual-Drive Flexible Knee Assistive Exoskeleton for Elderly Daily Locomotion

**DOI:** 10.34133/cbsystems.0576

**Published:** 2026-08-11

**Authors:** Shisheng Zhang, Yang Zhang, Yanzong Xu, Jinke Li, Yuquan Leng, Xinyu Wu

**Affiliations:** ^1^ Guangdong Provincial Key Lab of Robotics and Intelligent System, Shenzhen Institutes of Advanced Technology, Chinese Academy of Sciences, Shenzhen 518055, China.; ^2^ University of Chinese Academy of Sciences, Beijing 100049, China.; ^3^ Mechanical Industry Key Laboratory of Intelligent Robotics Technology for 3C Product and the Sino-German College of Intelligent Manufacturing, Shenzhen Technology University, Shenzhen 518118, China.; ^4^ MileBot Robotics Company Ltd., Shenzhen 518055, China.; ^5^ School of Biomedical Engineering, State Key Lab of Robotics and Systems, Harbin institute of Technology, Shenzhen 518055, China.

## Abstract

The knees, as important parts in daily locomotion, carry the body weight and provide stability and flexibility. With the degradation of muscle and endurance, the elders get weak knee strength, which heavily affects their life quality. To address this issue, a single-source dual-drive flexible knee assistive exoskeleton is explored to assist the elders in their daily locomotion. Firstly, a single-source dual-drive architecture is designed. The left and right knee are controlled by single electric motor with clutch. It reduces the weight of exoskeleton and has the potential to enhance interaction safety and comfort with cable-driven and elastic elements. Secondly, a cyclic locomotion pattern recognition (LPR) methodology for daily walking situation is provided, adopted with a dual detection strategy fused with finite state machine and fuzzy control. Daily walking situations, e.g., level walking (LW), ramp ascending (RA), ramp descending (RD), stair ascending (SA), and stair descending (SD), could be recognized accurately and precisely. Finally, a time-shared assistive torque control strategy with finite state is conducted to effectively provide suitable auxiliary torque in various walking environments based on locomotion patterns. Three young participants and 3 elder participants participate in outdoor LPR experiments in real-world environment and indoor energy loss experiments. The initial experiment results indicate that the average precision of LPR reaches 98.89%, and the average metabolic cost and surface electromyography signals reduce as much as 28.20% and 67.33%, respectively, with the assistance of exoskeleton.

## Introduction

As important parts in daily locomotion, the knees carry the body weight and provide stability and flexibility to effectively encourage daily walking [1], e.g., level walking (LW), ramp ascending (RA), ramp descending (RD), stair ascending (SA), and stair descending (SD). With the degradation of muscle strength and endurance, the elders experience weakened knee strength and insufficient body support, which severely affects their life quality and increases the falling risks [2]. According to the statistics [3], by the late 2070s, the global population of individuals aged 65 and above is expected to reach 2.2 billion, surpassing the number of children under 18 years of age. Researches on knee assistance exoskeleton preserve great importance on enhancing walking ability in daily environment for elderly people under the circumstances of world population aging.

Despite the development of various knee assistive exoskeletons [4–7], several challenges remain in providing effective and appropriate assistance strategies, specifically in enhancing the daily walking ability for elderly people. (a) The chosen drive methods do not fully consider the burden on elderly people; (b) The system cannot recognize and detect changes on cyclic locomotion pattern in real outdoor scenarios; (c) The existing devices only provide assistance for specific locomotion patterns, while the assistance strategy lacks the compatibility and flexibility to adapt the change of locomotion patterns in daily environment.

The knee assistive exoskeleton drive methods are classified into 3 types: motor-driven, pneumatic, and cable-driven systems. The motor-driven system directly binds the motor on the knee joint and provides joint movement by controlling the rotation of the motor [8–10]. However, this approach requires the motor mounted at the knee joint, which increases the weight and inertia of the whole exoskeleton system, leading extra burden to the wearer. The pneumatic system simulates the working procedures of human muscles and controls the movement of the exoskeleton by adjusting the air pressure [11,12]. It brings advantages in lightweight structure, flexible movement, compact size, pollution-free operation, and strong cushioning. However, with limitations of power, poor stability, low energy conversion efficiency, and requirement of an external air compressor as a driving source, the overall flexibility and portability of the pneumatic exoskeletons is constrained. In recent years, more and more explorations and researches have been published in cable-driven systems [13–15]. Cable-driven systems typically use Bowden cables or flexible ribbon cloth to transmit driving force, featuring simple and lightweight structures, strong flexibility, and excellent human compatibility and comfort. The cable-driven systems transmit force over long distance. Furthermore, the driving source could be mounted at the body’s center of gravity in order to reduce the burden when wearing the exoskeleton, hence enhancing the overall portability of the system. Based on the mentioned description, this study proposes a cable-driven system based on Bowden cable to provide assistance to the user’s knee joint.

To effectively implement knee assistive exoskeletons in daily life, the control system must accurately recognize various motion patterns and provide appropriate auxiliary torque based on specific motion modes. Several approaches have been developed for recognizing daily motion patterns. The simplest method involves manual switching [16,17]. While manual operation is effective and practical for gait pattern transitions especially for individuals with complete paraplegia, the user must accept training for operating wearable robots. This approach can be cumbersome for users who rely on crutches. In response, threshold- or rule-based methods have been introduced. Li and Hsiao-Wecksler [18] proposed a threshold-based automatic gait pattern recognition method for ankle-foot orthotics by using inertial measurement units (IMUs) to track the 3-dimensional position of the wearer’s legs. Yuan et al. [19] proposed a gait pattern recognition method based on fuzzy logic for prosthetic limb control, enabling the identification of gait patterns before the swing phase of the prosthetic limb. Recently, machine learning classification algorithms have also been applied for motion pattern recognition, including linear discriminant analysis (LDA) [17], vector analysis [20], and hidden Markov models [21]. However, these methods require extensive sets of training data for various motion patterns, which can impede their practical application [22]. Therefore, this study proposes a dual detection strategy combining finite state machine (FSM) and fuzzy control to identify the user’s current motion pattern effectively.

Additionally, sensors play a crucial role in the measurement, classification, and control of exoskeleton movement. Sensor systems for motion pattern recognition are broadly divided into 2 categories: bioelectric sensors and mechanical sensors. Bioelectric sensors include surface electromyography (sEMG) and electroencephalography (EEG). sEMG is frequently used to measure muscle activity during contractions via surface electrodes [23,24], while EEG detects and records electrical signals generated by cortical neuron activity [25]. However, electrodes used in bioelectric sensors must be directly attached to the limbs, making them easily affected by electrode position, noise, and skin condition. On the other hand, mechanical sensors are more robust to environmental factors and easier to embed in devices such as IMUs [9,26], depth cameras (D-RGB) [27,28], and light detection and ranging (LIDAR) [29] than bioelectrical sensors. Nevertheless, the depth cameras have limitations in the field of angle, resulting in sparse point clouds when the angle of the camera is too wide or too narrow. LIDAR may be too expensive to build a system. IMUs are widely used in various exoskeleton systems due to their high flexibility, accuracy, low noise, and reliability. Therefore, this study proposes a human lower limb motion pattern recognition system by fusing 4 IMU sensing signals.

To address the walking assistance requirements of the elderly in the daily environment and referenced with the analysis of existing studies, this paper presents the design of a novel single-source dual-drive (SSDD) flexible knee assistive exoskeleton (FKAE). The device employs a clutch to drive the left/right knee joint with a single motor, and it enhances interaction comfort and safety with cable-driven and elastic elements. Table 1 benchmarks this preliminary exploration of the proposed FKAE against existing systems. While traditional motor-driven [9] and cable-driven [13] systems improve joint actuation, they typically rely on heavy or complex multi-actuator configurations. Furthermore, existing locomotion pattern recognition (LPR) approaches often utilize bioelectric [23] or visual sensors [27], which suffer from physiological constraints or limited fields of view. In contrast, our design explores a novel SSDD architecture utilizing robust IMUs. Integrated with a computationally efficient Fuzzy-FSM, the system achieves smooth locomotion transitions while avoiding the high computational costs of data-intensive machine learning models. The main contributions of this paper are listed as follows:

**Table 1.** Comparison of the proposed FKAE with existing exoskeleton systems

Reference	Type and actuation	Sensor	Strategy	Advantages	Limitations
Liu et al. [9]	Rigid knee, motor-driven	IMU + Encoders	Threshold-based FSM	High control bandwidth	Heavy distal mass and high inertia
Wang et al. [13]	Hybrid knee, dual cable	IMU + Encoders	Gait phase estimation	Excellent compliant interaction	Dual motors increase weight and complexity
Luo et al. [23]	Hip exo., motor-driven	sEMG	ML (dual-task)	High accuracy in lateral gaits	sEMG sensitive to noise and skin conditions
Wang et al. [26]	Hip exo., motor-driven	D-RGB camera	Neural network	Anticipatory adaptation	Limited FOV and sparse point clouds
Proposed FKAE	Flexible knee, single-motor	Four IMU fusions	Fuzzy-FSM integration	Lightweight, robust sensing, smooth transitions	Predefined torque profiles (exploratory)

1. This paper designs an SSDD knee assistive exoskeleton prototype. It adopts a single motor with cable-driven and elastic elements to drive the joint, while a clutch mechanism allows the motor driving both the left and right knee joints. This design approach reduces the overall weight of the exoskeleton system and improves the flexibility of the driving mechanism, which further reduces the load bearing burden of the user and improves the safety of interaction.

2. A dual detection scheme for locomotion patterns in daily life is proposed. It adopts fuzzy control to recognize the user’s locomotion pattern in steady-state mode and FSM algorithm to detect the changes in locomotion pattern during mode transitions. This scheme can accurately identify common daily motion scenarios such as LW, RA, RD, SA, and SD while also reducing detection delays during motion mode transitions.

3. A finite state time-sharing auxiliary control strategy is developed. The strategy provides a torque assist configuration that matches the user’s current motion pattern. The experimental results indicate that the average metabolic cost and the rate of change (ROC) of sEMG signal are reduced by 28.20% and 67.33%, respectively, with the deployment of exoskeleton based on this auxiliary control strategy.

The main research content of this paper includes the structural design of a flexible knee assist exoskeleton, LPR algorithms, assistive torque control strategies, and experimental validation. The structure of this paper is as follows. Materials and Methods introduces the hardware design of the knee assistive exoskeleton, the LPR method, and the finite state time-sharing auxiliary control strategy. The experimental settings and results are presented and described in Experimental and Results. Discussion presents the discussion, and conclusions will be drawn in Conclusion.

## Materials and Methods

This section mainly introduces the mechatronic design of the SSDD FKAE, a cyclic motion pattern recognition method based on a dual recognition strategy, and a finite state time-shared assistive torque control strategy based on the recognized locomotion patterns, used in this study.

### Mechatronics design

**Fig. 1.**
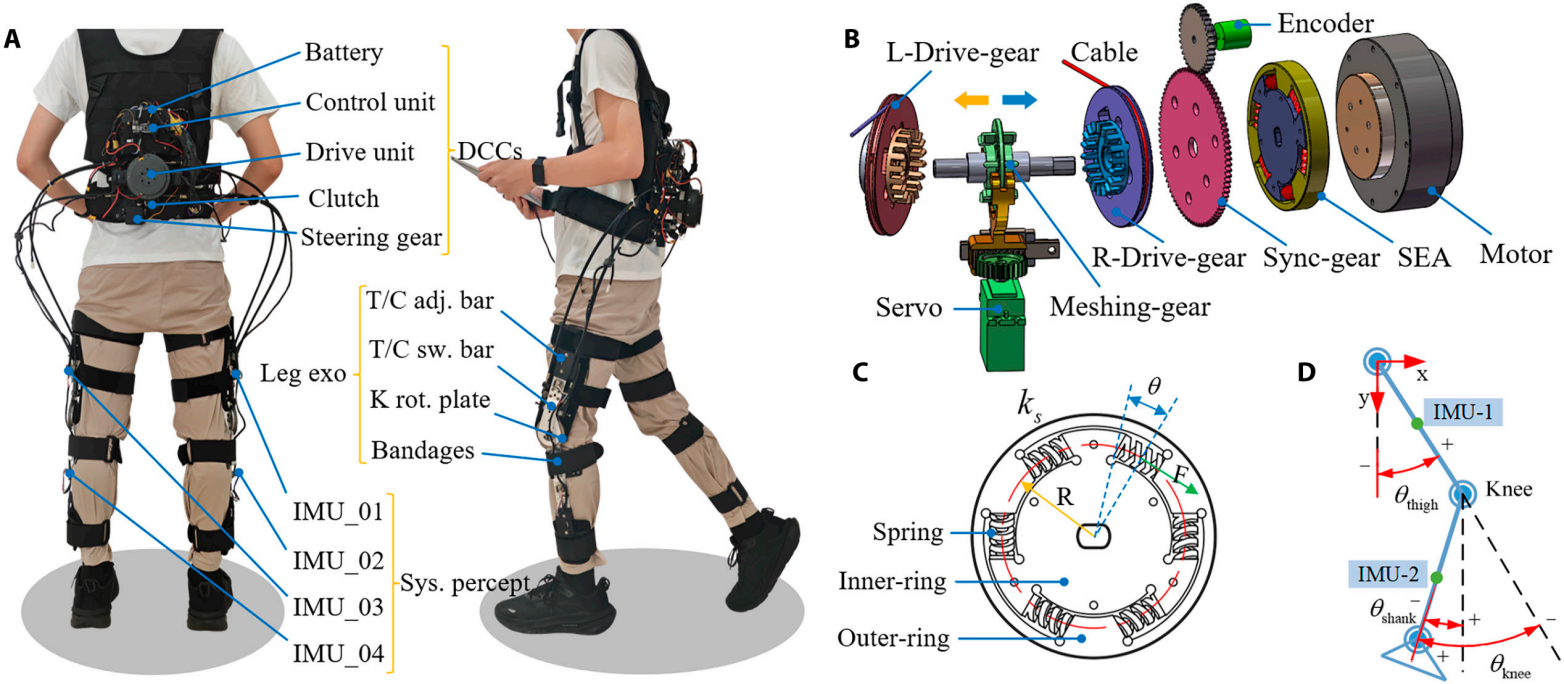
The working principle of the knee assistive exoskeleton FKAE. (A) The prototype of the single-source dual-drive flexible knee assistive exoskeleton. (B) Diagram of the actuator structure and its working principles. The yellow arrow indicates that the moving mesh gear is engaged with the left-side drive gear; the blue arrow indicates that the moving mesh gear is engaged with the right-side drive gear. (C) Disc-type series elastomers based on linear springs. (D) Schematic diagram of the correspondence between the IMU layout and knee angle solution. DCCs, drive control components.

The SSDD FKAE designed in this paper is presented in Fig. 1A. It consists of 3 main components: the drive control components (DCCs), the bilateral lower leg exoskeleton, and locomotion pattern sensing system. The drive control module includes the control unit, drive unit, battery, steering gear, clutch mechanism, etc. The bilateral leg exoskeleton is composed of thigh/calf adjusting rod, thigh/calf swinging rod, knee joint rotating discs, and binding straps, etc. The locomotion pattern sensing system consists of 4 IMU sensors mounted on the FKAE’s thigh and calf swinging rods.

Previous studies [30] have shown that placing the joint module and drive unit of the device at the waist results in lower energy loss during user movement than placing them at the joints. Therefore, the drive control of the FKAE is mounted on the user’s back, with a Bowden cable system connecting it to the bilateral lower limb exoskeleton to assist the knee joint. The Bowden cable allows certain and appropriate bending when transmitting kinetic energy, enabling it to bypass obstacles and automatically compensate the displacement differences caused by the relative movement between the lower limbs and the user’s back during activity, thus ensuring the effectiveness of FKAE’s assistance. The total weight of the FKAE is approximately 3.2 kg, with each unilateral lower limb exoskeleton weighing about 0.7 kg. The weight of the control electronics and battery is around 0.8 kg, and the rest of the mass is mainly shoulder straps and binding structures.

The FKAE is designed to improve the walking ability of elderly people and includes the following features: (a) SSDD: this scheme is leveraged to provide time-sharing assistance for the user’s left/right knee, leading to the less adoption of high-power drive motor and thus decreasing the overall weight of the whole system; (b) series elastic drive: an elastic component is attached between the drive motor and the load, which alleviates the rigid impact caused by sudden state changes of the cable or accidental falls of the user.

#### SSDD actuation

FKAE adopts the SSDD mechanism. When the knee joint needs assistance on one side, the torque transmission path between the drive motor and the knee joint is established. Meanwhile, the torque transmission path between the drive motor and the knee joint is interrupted on the other side. This innovative approach ensures the targeted assistance without cross-interference, optimizing the performance of the entire system. The FKAE system has a torque control bandwidth of 12.3 Hz (−3 dB), which can meet the movement frequency of the human knee joint (usually 2 to 3 Hz).

A clutch structure is designed in this paper based on the SSDD drive mechanism. This design is composed of output shaft, left and right drive gears, moving meshing gear, and a servo motor, as presented in Fig. 1B. The servo motor controls the axial movement of the moving meshing gear in order to mesh with the left/right driving gear through the gear-rack mechanism. Thus, the output torque of the driving motor is controlled on purpose. The left and right drive gears attach a Bowden cable to the leg exoskeleton, transmitting the rope-driven torque to the leg exoskeleton. For instance, when the FKAE provides assistance to the left knee joint, the servo motor strategically moves the moving meshing gear to mesh with the left drive gear, thereby directing the motor’s torque to the left drive gear exclusively. Identically, when the right knee joint requires assistance, the servo motor disconnects the mobile meshing gear from the left and moves to the position where it engages the right drive gear, ensuring appropriate assistance for both joints.

Meanwhile, an elastomer is embedded between the motor’s output and the load to form series elastic actuator (SEA) structure [31–33] to ensure the flexibility and interaction safety of the mechanism from the hardware. The user’s daily activities are random. When encountering obstacles or accidental falls, the user stops body movement immediately, which inflicts abrupt torque against the drive motor. SEAs are connected in series between the driver source and the load, enhancing the flexibility and impact resistance of the system. A drive motor (ME18-A, Dogtex, Shenzhen) was selected to drive the SEA. The planetary gear ratio is only 6:1, which can achieve low impedance drive and reduce noise. After transmission, the SEA can provide 8 N‧m continuous torque and 18 N‧m peak torque at a velocity of up to 350 r/min.

The SEA designed in this study takes the linear spring as foundation. As presented in Fig. 1C, the structure consists of a linear spring, an inner ring, and an outer ring structure. Specifically, the linear spring is a high-stiffness rectangular spring. The elastic outer ring is fixed on the output shaft of the driving motor by bolts. Thus, the output torque of the driving motor is transmitted to the elastic inner ring through the rectangular spring. Given the minor relative rotational displacement between the elastic inner ring and the outer ring, the compression of the internal spring is approximated as a linear process.

The torque generated by the spring is calculated via the following equation:

$$T=3{\mathrm{Rk}}_{s}\delta$$ (1)

When the relative rotation angle θ is small, the compression of the rectangular spring can be approximately considered as:

δ=Rsinθ≈Rθ (2)

Therefore, the theoretical stiffness *k* of the disc-type elastomer is approximately calculated by the following equation:

$$k=\frac{T}{\theta }=3k_{s}R^{2}$$ (3)

where θ represents the relative rotation angle between the elastic inner ring and the outer ring, δ represents the linear compression amount of the rectangular spring, $k_{s}$ represents the stiffness of the rectangular spring, and *R* represents the magnitude of the force arm generated by the spring force *F*.

FKAE is designed with the application of compliant actuators, as illustrated in Fig. 1B. The compliant actuator is developed with reference to the concept of the SEA [32], which installs elastic elements between the driving motors and the robot joints. The motor drives the rope coiled around a spool, which pulls the curved spring via a rotary table, transmitting motion to the output rod and ultimately rotating the robot joint. The compliant actuator can be functionally equated to a torque sensor, where the output torque/force can be obtained by simply measuring the deflection of the elastic element (usually with encoder readings at both ends of the element) and using that torque as state feedback for a finite state time-shared assistive torque control strategy for real-time adjustment of the torque output of the FKAE. Additionally, a proportional-integral-derivative (PID) control scheme is proposed to drive FKAE to track such torque, which guarantees a safe interaction and also encourages the user to execute voluntary efforts and hence speed up the recovery. This design balances compact size, lightweight construction, high-density output, and low cost, providing a guarantee for practical implementation. In addition, previous studies have shown [34] that the introduction of physical elastic elements between rigid actuators and external loads can not only improve the compliance of human–robot interaction but also partially mask the friction and reflex inertia in the motor and transmission mechanism. The back driving torque of the system is 0.25 N‧m, and the reflected inertia of the system is 0.0024 kg m^2^, under unpowered conditions. Taking the Bowden cable loss into account, the average interaction torque of the FKAE system was measured to be 0.052 N‧m under zero-torque control condition, which also demonstrated the advantages of the designed system in minimizing the undesirable resistance and ensuring the good transparency of the system.

The actuator’s 18 N‧m peak torque is strategically designed to provide 21% to 25% of the biological demand for high-effort tasks like SA (70 to 84 N‧m for a typical 70-kg adult). This partial assistance represents an optimal engineering trade-off prioritizing human–robot compliance. Specifically, a low 6:1 planetary gear ratio restricts absolute torque but successfully minimizes reflected inertia and unpowered back-driving torque to just 0.25 N‧m. Furthermore, while Bowden cables inherently introduce friction, the SEA structure and PID control actively mask this resistance, yielding an exceptional zero-torque interaction of 0.052 N‧m. Ultimately, this design balances sufficient assistive torque with low impedance and high transparency.

#### Locomotion mode perception system

Four IMUs are integrated in the proposed system to build the human lower limb LPR strategy. As illustrated in Fig. 1D, the IMU sensors are mounted in the middle of the thigh and calf component of FKAE. The joints of the lower limbs of the human body perform notable movement regularity on the sagittal plane in daily activities. The performed regularities provide reference for designing motion pattern recognition strategy. To facilitate the calculation of the knee joint angle on the sagittal plane, it is critical to ensure that *x*–*y* plane of the IMU keeps parallel to the human sagittal plane and perpendicular to the horizontal plane.

In Fig. 1D, $\theta_{\text{thigh}}$ represents the angle between the *y* axis in the sagittal plane of the IMU at the thigh and the vertical axis of the human body, which is further addressed as the thigh angle. $\theta_{\text{shank}}$ represents the angle between the *y* axis in the sagittal plane of the IMU at the shank and the vertical axis of the human body, which is further addressed as the shank angle. The thigh angle and shank angle are set to zero when locating at the vertical axis position. The angle of knee joint is further calculated by the following equation:

$$\theta_{\text{knee}}=\theta_{\text{thigh}}-\theta_{\text{shank}}$$ (4)

where $\theta_{\text{thigh}}$ and $\theta_{\text{shank}}$ are calculated with a signed operation in Eq. (4), and the sign of the value depends on the relative pose between the limb and the coronal plane. Since this method ignores the movement of human limbs on the coronal plane, it is required to calibrate the position angle to zero after the user wears FKAE appropriately. Angles of all components are set to zero taking the user’s natural standing state as references. The IMU (JY901S, Taidacent) reads the original data of the sensor, such as the 3-axis acceleration, 3-axis angular velocity, and 3-axis magnetic field, and uses the attitude dynamic core algorithm combined with the high-dynamic Kalman filter fusion algorithm to solve the real-time stable 3-axis attitude angle. Additionally, the magnetic field sensor filtering fusion is added to the *z*-axis heading angle, which effectively solves the cumulative error caused by the drift of the gyroscope in the 6-axis algorithm, and can stably output heading angle data over the long term. The IMU’s high sampling rate (200 Hz) ensures that latency remains acceptable in the real-time control loop and does not significantly impact the FKAE’s response time.

The system needs to monitor the user’s current motion state in real time to ensure the user’s safety when walking with the help of FKAE. First, when the system detects that the absolute values of the joint angles/angular velocities collected by all IMUs are less than a threshold, the system determines that the user is in a falling/standing state, and FKAE stops assisting. Second, when the system detects that the motor is in a stalled state, it determines that the system is in an abnormal state and the motor stops working.

### Locomotion mode recognition method

To provide effective assistance to users, the FKAE exoskeleton must automatically recognize the user’s movement intentions and patterns. In daily life, users engage in cyclic activities composed of various motion modes with FKAE worn on. This process is categorized into steady-state mode and toggle-state mode. The steady-state mode refers to the user moving through multiple consecutive gait cycles within the same scenario, while the toggle-state mode involves transitioning from one motion scenario to another.

This paper introduces a cyclic LPR scheme utilizing a dual recognition strategy, incorporating fuzzy control and a FSM algorithm. The scheme consists of 3 aspects: calibration of detection points and locomotion data acquisition, steady-state pattern recognition, and toggle-state pattern recognition. The calibration of detection points and locomotion data acquisition phase obtains the user’s motion data in various motion modes via IMU sensors and establishes the triggers for key points in LPR. For steady-state pattern recognition, fuzzy control is employed to identify motion patterns within steady states. In toggle-state mode, the FSM algorithm is utilized to recognize transitions between different motion patterns.

#### Calibration of detection point and locomotion data collection

**Fig. 2.**
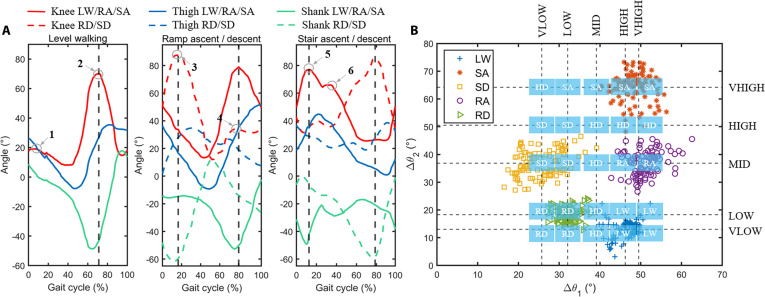
Kinematic characteristics of users in different motion modes. (A) Angle graphs of various components during level walking, ascending/descending stairs, and ascending/descending slopes. (B) Distribution patterns of variables $\Delta \theta_{1}$ and $\Delta \theta_{2}$ across different motion modes. VLOW, very low; LOW, low; MID, medium; HIGH, high; VHIGH, very high.

In various motion patterns, a unilateral knee joint performs multiple peaks within a single gait cycle. However, the calf and thigh angles only exhibit an increasing trend at the maximum peak. This timing of the maximum knee joint angle is referred as the knee joint characteristic peak, as illustrated in Fig. 2A. Utilizing the presence of knee joint characteristic peaks across different motion modes and the specific arrangement of sensors on the assistive knee exoskeleton, this paper proposes a detection scheme where the knee joint characteristic peak performs as the key trigger for LPR.

As presented in Fig. 2B, the raw motion data were collected by users performing multiple gait cycles during walking with FKAE exoskeleton worn on in the following 5 scenarios: LW, ascending/descending stairs, and ascending/descending ramps. Data from 50 gait cycles per scenario were collected to calculate the average joint trajectories. Additionally, by analyzing the motion data of the FKAE in different usage scenarios, the differences in movements across these scenarios were identified in the subsequent construction of the motion pattern recognition algorithm. $\Delta \theta_{1}$ and $\Delta \theta_{2}$ represent the range of the thigh within a single cycle and the difference between the maximum values of the thigh and calf within a single cycle, respectively.

#### Fuzzy control based for steady-state mode recognition

Fuzzy control [35,36] is a set theory method used to handle scenarios with ambiguity, uncertainty, or with implicit boundary. Fuzzy control allows elements performing partial membership rather than just binary states of belonging or not belonging in traditional set theory. The core concept of fuzzy control is to describe the relationship between elements and fuzzy sets through membership functions. A membership function quantitatively describes fuzzy concepts, mapping elements to a value within the interval of [0,1], indicating the degree to which they belong to a fuzzy set.

**Fig. 3.**
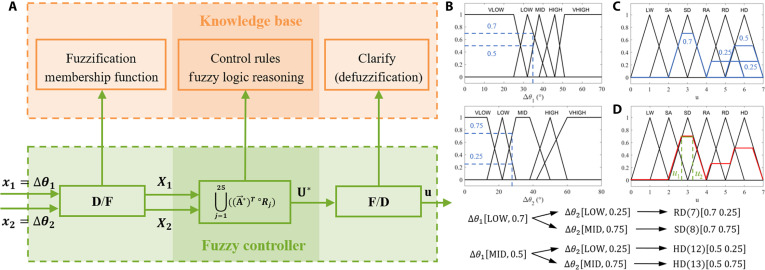
Schematic diagram of steady-state motion pattern recognition method constructed with a fuzzy controller. (A) Construction of the fuzzy controller. (B) Fuzzification for input variables. (C) Calculation of fuzzy outputs based on the fuzzy rule base and fuzzy logic reasoning. (D) Schematic diagram of de-fuzzification processing based on maximum membership degree method (mean value).

This paper develops a steady-state LPR method based on a Mamdani-type fuzzy controller [37], consisting of 3 aspects: (a) feature acquisition and fuzzification: the input features are derived from the motion data collected by IMU sensors, and appropriate membership functions are designed to fuzzify the input features; (b) establishment of fuzzy rules and fuzzy logic reasoning: a reasonable set of fuzzy rules is developed and an appropriate fuzzy logic reasoning method is selected to convert the fuzzified input features into corresponding fuzzy output motion patterns; (c) defuzzification: the fuzzy motion patterns are converted into clear and precise LPR results through defuzzification, as presented in Fig. 3A.

1. Feature acquisition and fuzzification: Fuzzy logic is applied at each characteristic peak moment of the knee joint for motion pattern recognition. At this moment, the feature variables $\Delta \theta_{1}$ and $\Delta \theta_{2}$ are calculated for each detection cycle. These variables perform as input variables for fuzzy logic, calculated using the following equations:

$$\begin{array}{l} \Delta \theta_{1}=\theta_{\text{thigh}\_\text{max}}-\theta_{\text{thigh}\_\text{min}}\\ \Delta \theta_{2}=\theta_{\text{thigh}\_\text{max}}-\theta_{\text{shank}\_\text{max}} \end{array}$$ (5)

where $\theta_{\text{thigh}\_\text{max}}$ represents the maximum peak value of the thigh angle in a single cycle, $\theta_{\text{thigh}\_\text{min}}$ represents the minimum value of the thigh angle in a single cycle, and $\theta_{\text{shank}\_\text{max}}$ represents the maximum value of the calf angle in a single cycle.

Fuzzification involves mapping the distinct values of input/output to fuzzy subsets based on membership functions. Before fuzzification, it is necessary to determine the range of input variables. As presented in Fig. 2B, the range of input variable $\Delta \theta_{1}$ within a gait task detection cycle is $\Delta \theta_{1}\in \left[0^{\circ },\;70^{\circ }\right]$, and the range of $\Delta \theta_{2}$ is $\Delta \theta_{2}\in \left[0^{\circ },\;80^{\circ }\right]$ based on the distribution pattern of the collected motion data. Fuzzy subsets are defined subsequently including very low (VLOW), low (LOW), medium (MID), high (HIGH), and very high (VHIGH). For input variable $\Delta \theta_{1}$, trapezoidal membership functions are selected for very low (VLOW) and very high (VHIGH). The input variable $\Delta \theta_{2}$ for very low (VLOW), medium (MID), and very high (VHIGH) also adopts a trapezoidal membership function, while other fuzzy subsets use triangular membership functions. The output variable *u*, representing the motion pattern, is divided into 6 levels, taking number 1 for LW, number 2 for SA, number 3 for SD, number 4 for RA, number 5 for RD, and number 6 for Hold (HD). Triangular membership functions are selected for all the motion modes. The detailed division results are presented in Fig. 3B.

2. Establishment of fuzzy rule base and fuzzy logic reasoning: The fuzzy rule base can be developed by observing and analyzing the distribution patterns of the input variables $\Delta \theta_{1}$ and $\Delta \theta_{2}$ in different motion modes, as presented in Fig. 2B. The clustering patterns of the variables $\Delta \theta_{1}$ and $\Delta \theta_{2}$ in differnent motion modes indicate distinct groups, leading to the summarization of the fuzzy rule base listed in Table 2. Each fuzzy rule involves an implication relationship $R_{i}\left(i=1,\cdots ,25\right)$, forming the overall system of implications $R=R_{1}\cup R_{2}\cup \cdots \cup R_{25}$.

**Table 2.** Base of fuzzy rules

*u*		$\Delta \theta_{1}$				
		VLOW	LOW	MID	HIGH	VHIGH
$\Delta \theta_{2}$	VLOW	RD (1)	RD (6)	HD (11)	LW (16)	LW (21)
	LOW	RD (2)	RD (7)	HD (12)	LW (17)	LW (22)
	MID	SD (3)	SD (8)	HD (13)	RA (18)	RA (23)
	HIGH	SD (4)	SD (9)	HD (14)	HD (19)	HD (24)
	VHIGH	HD (5)	SA (10)	SA (15)	SA (20)	SA (25)

Fuzzy logic reasoning refers to the process of converting system input into output by using the established fuzzy rule base. Due to the design of membership functions and rule base, a set of input variables activates several rules in the rule base. In order to establish the relationship between these activated rules, the overall output of the fuzzy logic reasoning is calculated via Eq. (6). This process is represented using the graphical method (such as $\Delta \theta_{1}=35,\Delta \theta_{2}=28$), where the corresponding graph of the rule to be activated is superimposed, as presented in Fig. 3C.

$$U^{*}=\overset{25}{\underset{j=1}{\cup }}\left({\left({\vec{A}}^{*}\right)}^{T}\cdot R_{j}\right)$$ (6)

where $U^{*}$ represents fuzzy set and $R_{j}$ represents the *j*th relation matrix.

3. Defuzzification: The output of fuzzy reasoning is a fuzzy set in a fuzzy state, which lacks physical meaning until it undergoes a defuzzification process to yield a crisp value (CV) with practical significance. At this stage, the fuzzy set U can be understood as an irregular shape formed by the superposition of multiple simple shapes, where precise calculations are further required being applied to the overlapping areas. The fuzzy output generated by the Mamdani method is a membership function curve, describing how the fuzzy values of the output variable change across the output range. The maximum membership method (mean value) is a common defuzzification technique, where the final crisp output is the average of the horizontal coordinate at the points of maximum membership on this function curve. The specific calculation is presented as the following equation:

$$u=\frac{u_{1}+u_{2}}{2}$$ (7)

where *u* represents the output of the horizontal coordinate and $u_{1},u_{2}$ represent the value of the horizontal coordinate at the maximum membership. The principle of the maximum membership method (mean value) is illustrated in Fig. 3D. The red solid line in the figure represents the membership function curve of the fuzzy set derived from Mamdani reasoning. The green dashed line indicates the horizontal coordinate values at the maximum membership on the membership function curve. The average of these *x* coordinates along the *x* axis is the defuzzified output value CV.

#### FSM based for toggle-state mode recognition

The steady-state pattern recognition algorithm based on fuzzy control contains the following defects during the toggle between motion modes: (a) high detection delay, where fuzzy control cannot timely detect changes in the actual motion modes, requiring several gait cycles after the mode transition to identify the new motion mode; (b) mode misjudgment, where fuzzy control sometimes identifies the wrong motion mode during toggles, also requiring several gait cycles after transition to recognize the correct motion mode.

**Fig. 4.**
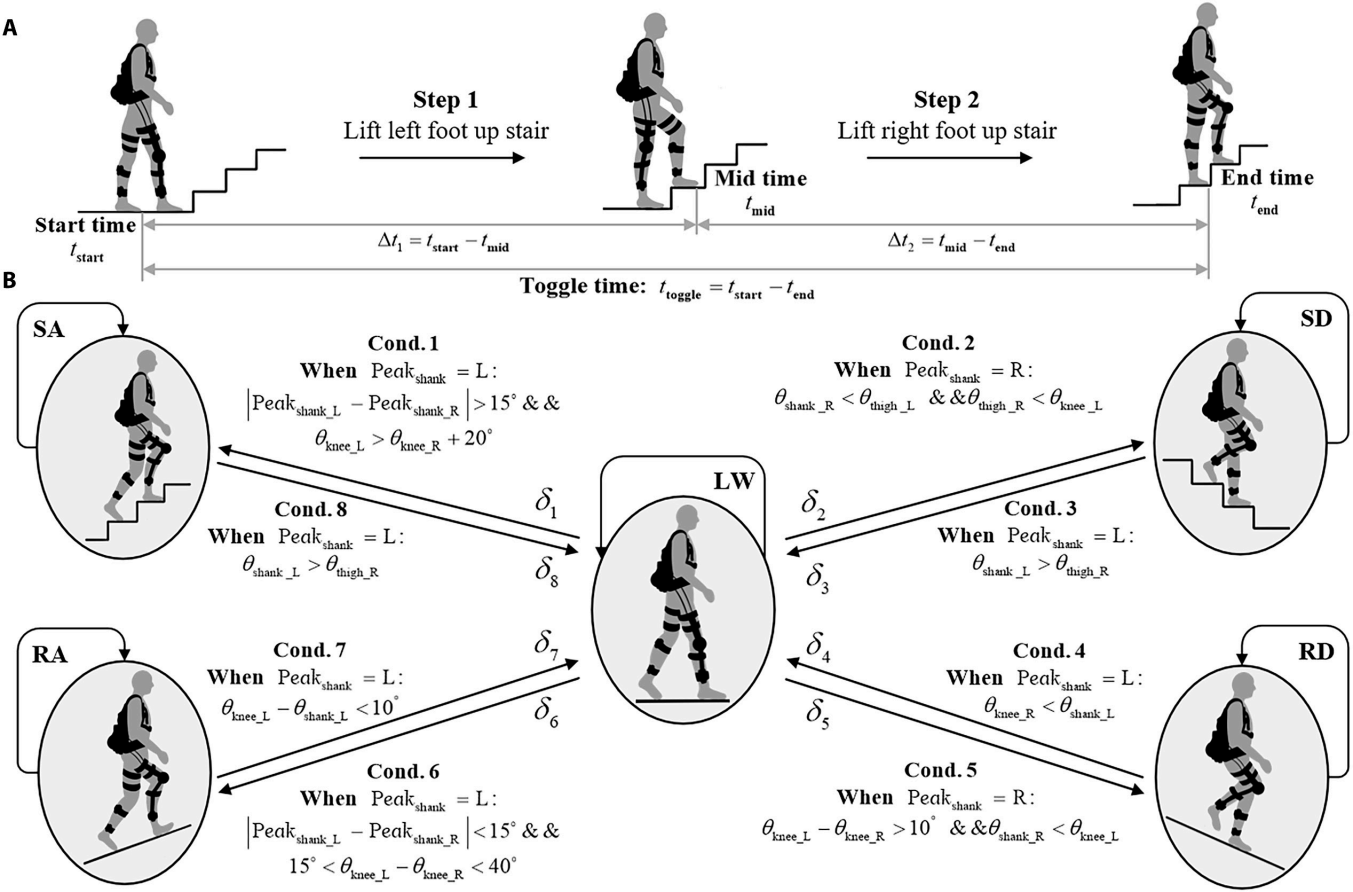
Definition of toggle-state mode process and state transfer condition diagram between different motion modes. (A) Process of transitioning from level walking to ascending stairs. (B) Finite set of transition states formed between level walking, ascending/descending stairs, and ascending/descending slopes. The variables ${\text{Peak}}_{\text{shark}}=L$ and ${\text{Peak}}_{\text{shark}}=R$ indicate the moments when the left and right shank angles reach their peaks, while ${\text{Peak}}_{\text{shark}}$ and ${\text{Peak}}_{\text{shark}}$ represent the peak angles of the left and right shanks, respectively. LW, level walking; SA, stair ascent; SD, stair descent; RA, ramp ascent; RD, ramp descent.

Therefore, there is a need to enhance the system’s detection speed during mode transitions and improve the accuracy of the pattern recognition algorithm. An FSM [38–40] is a mathematical model with discrete input and output, possessing multiple internal states and enabling transitions between these states based on conditional judgments. FSM is essentially a quintuple of 5 internal parameters $M=\left[I,\;O,\;S,\;\delta ,\;F\right]$: event input, event output, a set of internal states, state transition conditions, and action output functions, where:

• $I=[i_{1}=\theta_{\text{knee}\_L},i_{2}=\theta_{\text{knee}\_R},i_{3}=\theta_{\text{thigh}\_L},i_{4}=\theta_{\text{thigh}\_R},$ $i_{5}=\theta_{\text{shank}\_L},i_{6}=\theta_{\text{shank}\_R}]$ represents the input signals, where $\theta_{\text{knee}\_L}$ and $\theta_{\text{knee}\_R}$ represent the left and right knee joint angle, respectively, $\theta_{\text{thigh}\_L}$ and $\theta_{\text{thigh}\_R}$ represent the left and right thigh angle, respectively, and $\theta_{\text{shank}\_L}$ and $\theta_{\text{shank}\_R}$ represent the left and right shank angle, respectively;
• $O=\left[o_{1}=T,\;o_{2}=F\right]$ indicates the output signal, and T/F indicates that the user’s motion mode has/has not changed, respectively;
• $S=\left[s_{\mathrm{LW}},\;s_{\mathrm{SA}},\;s_{\mathrm{SD}},\;s_{\mathrm{RA}},\;s_{\mathrm{RD}}\right]$ represents the set of 5 motion mode states, including LW, SA, SD, RA, and RD;
• $\delta =\left[\delta_{1},\;\delta_{2},\;\delta_{3},\cdots ,\;\delta_{8}\right]$ represents the state transition function, namely, the state transition condition, including 8 kinds of motion mode switching conditions, as presented in Fig. 4B for the specific conditions;
• *F* represents the output function that outputs the user’s current motion mode identified by the FSM.

Human daily activity is essentially a continuous action set composed of multiple same gait tasks or associated gait tasks. Once a current gait task is identified or determined, the probabilities of the subsequent gait tasks can be estimated based on the observed patterns and context. For example, if the current gait task involves ascending stairs, the next gait task is highly possible of either continuous ascending stairs or transition to flat walking, rather than descending stairs or other unrelated gait tasks. Therefore, based on the known motion patterns and transition conditions, the recognition of transition state motion patterns can be effectively identified with the FSM algorithm.

Toggle-state mode refers to the process where a user transitions from one steady-state motion scenario to another. Fig. 4A illustrates the process of a user switching from LW to ascending stairs. The moment when one foot begins to leave the ground is marked as the start of the mode transition $t_{\text{start}}$. The middle of the transition is noted as $t_{\mathrm{mid}}$ when one foot is on the ground and the other on the stairs, and the transition ends when both feet are on the stairs, which is marked as $t_{\mathrm{end}}$. The duration from the start to the end of the motion transition is marked as the transition time $t_{\text{toggle}}$, indicating the transition from LW to ascending stairs. Similar principles apply for other motion mode transitions. The 5 motion modes including LW, ascending/descending stairs, and ascending/descending slopes form a finite state set with 8 possible transition states. To accurately identify the specific conditions for transitions between different states, a detailed analysis of the kinematic characteristics during different motion mode transitions is necessary, as presented in Fig. 4B.

### Assistive torque control based on LPR

**Fig. 5.**
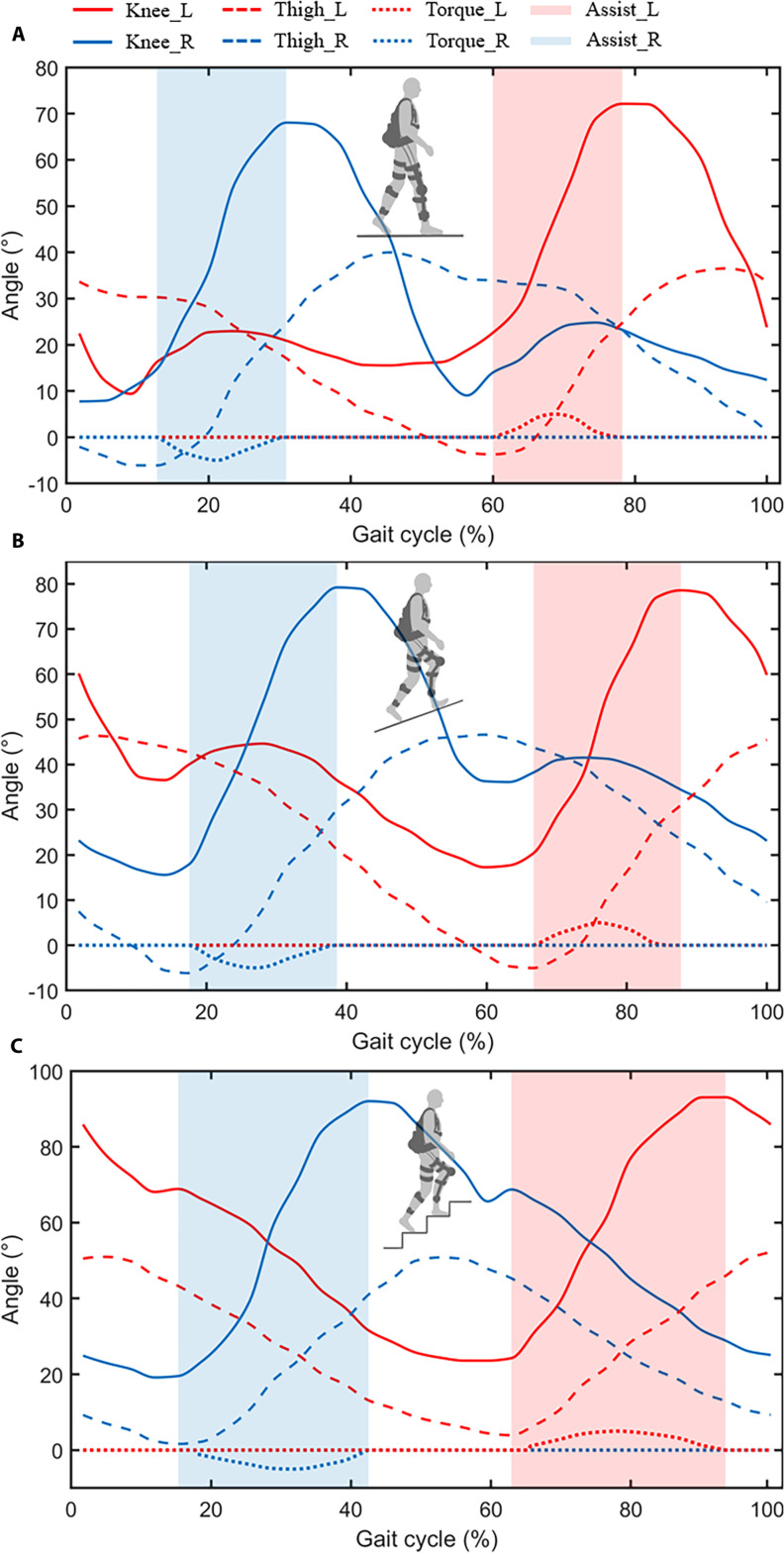
Schematic diagram of finite state time-sharing auxiliary control strategy. Scenarios of level walking (A), ascending a slope (B), and ascending stairs (C).

Based on the recognized locomotion patterns, this paper proposes a finite state time-shared assistive torque control strategy, where the FKAE provides external torque support to the user’s knee joints. Based on the daily activity rules, this study provides appropriate auxiliary strategies for 3 locomotion modes: LW, ascending ramps, and ascending stairs, as presented in Fig. 5. Functionally, the stepping cycle during SA can be divided into 3 periods: weight acceptance (WA), single limb support (SLS), and swing limb advancement (SLA). During early SLA, increasing plantar flexion activity in the trailing lim helps transfer body weight to the lead limb, while increasing biceps femoris and gastrocnemius activity facilitates knee flexion. As the foot lifts from the stair, biceps femoris and tibialis anterior activation increases rapidly to ensure adequate flexion of the knee and ankle to clear the risers. Activation of the semitendinosus in late SLA prepares the limb for the demands of WA [1]. The time-sharing assistance strategy relies on the coordination between the clutch and the drive motor, which provides assistance when the knee joint performs substantial flexion and stops assistance at the peak of the flexion. When the drive motor stops working, the clutch switches to the other side, preparing to assist the other knee joint. The final effect is illustrated in Fig. 5, and the direction of the torque is indicated by positive and negative signs. Meanwhile, the motor’s rotation direction is represented with clockwise as positive.

The power output torque curve designed in this paper is related to the real-time angle of the user’s knee joint [41,42]. When the LPR controller identifies that the current motion mode of the user is LW, ascending stairs, or ascending a slope, the controller determines the user’s assistance range and generates the expected torque curve according to the knee joint’s flexion angle. The knee joint is crucial for efficient movement, supporting body weight, absorbing shocks, and providing foot clearance. The angle and torque of the knee joint provide a comprehensive description of its action and functionality. The establishment of torque–angle relationship of knee joint is a commonly used measurement method to characterize knee joint stiffness [4], which is essentially to model knee joint torque as torsional configuration at different stride stages. FKAE continuously collects motion data from the user, recording the knee joint angle characteristics and determining the peak flexion angles of the user’s knee joints. By detecting key gait task events, the initial flexion angle of the user’s knee joint is identified. Three critical control points of the expected torque curve are determined by the preset peak point of the system and the characteristic angle of the start/end of the assistance. The assistive torque matching the real-time angle of the user’s knee joint is calculated according to the torque curve equation:

$$M_{d}\left(\theta \right)=\left\{\begin{array}{ll} K_{n}\;\text{sin}\left(\frac{\pi \left(\theta -\theta_{s}\right)}{3\left(\theta_{\mathrm{mid}}-\theta_{s}\right)}\right) & \theta_{s}\leq \theta <\theta_{\mathrm{mid}}\\ \frac{-K_{n}}{\theta_{e}-\theta_{\mathrm{mid}}}{\left(\theta -\theta_{\mathrm{mid}}\right)}^{2}+K_{n} & \theta_{\mathrm{mid}}\leq \theta <\theta_{e} \end{array}\right.$$ (8)

where $M_{d}\left(\theta \right)$ represents the magnitude of the expected torque value, θ represents the current knee joint angle, $\theta_{s}$ indicates the knee joint angle when assistance begins, $\theta_{\mathrm{mid}}$ represents the angle at the assistance torque reaching its peak value, $\theta_{e}$ indicates the angle when assistance stops, and $K_{n}$ represents the gain coefficient of the assistive torque. Take the left knee joint as an example: $\theta_{s}$ is defined as the minimum angle of the left thigh; $\theta_{e}$ is defined as the maximum flexion angle of the left knee joint in the previous cycle (if the left knee joint has reached the maximum flexion angle in advance in the current gait cycle, the assistance will be stopped in advance); $\theta_{\mathrm{mid}}$ is defined as the middle angle between the starting angle and the ending angle. The right knee joint is similar.

## Experimental and Results

### Experimental setup

A total of 6 participants, i.e., 3 healthy adults and 3 elderly male (4 males and 2 female), participated in the experiment. Their body feature parameters are presented in Table 3. Participants were randomly recruited without specific requirements for body type (such as height and weight), since the exoskeleton can be adjusted to fit various sizes. However, due to the limitations of the FKAE hardware conditions, if the device is to provide effective assistance to the user, the user’s body feature parameters (height: 1.50 to 1.85 m and weight less than 80 kg) must be restricted accordingly. All participants were asked to wear the FKAE exoskeleton and walk on different terrains (LW, SA, SD, RA, and RD) to preliminarily explore the feasibility and effectiveness of LPR methods and finite state time-sharing auxiliary control strategy. All participants provided written consent before the experiment. Approval of all ethical and experimental procedures and protocols was granted by the Medical Ethics Committee of Shenzhen Institutes of Advanced Technology (SIAT-IRB-250915-H1008).

**Table 3.** Statistical data of the participants

Subjects	Sex	Age/years	Height/m	Weight/kg
#1	Female	20	1.60	51
#2	Male	21	1.73	63
#3	Male	21	1.67	58
#4	Male	66	1.62	60
#5	Male	60	1.66	65
#6	Female	57	1.53	54

#### Locomotion mode recognition experimental setup

**Fig. 6.**
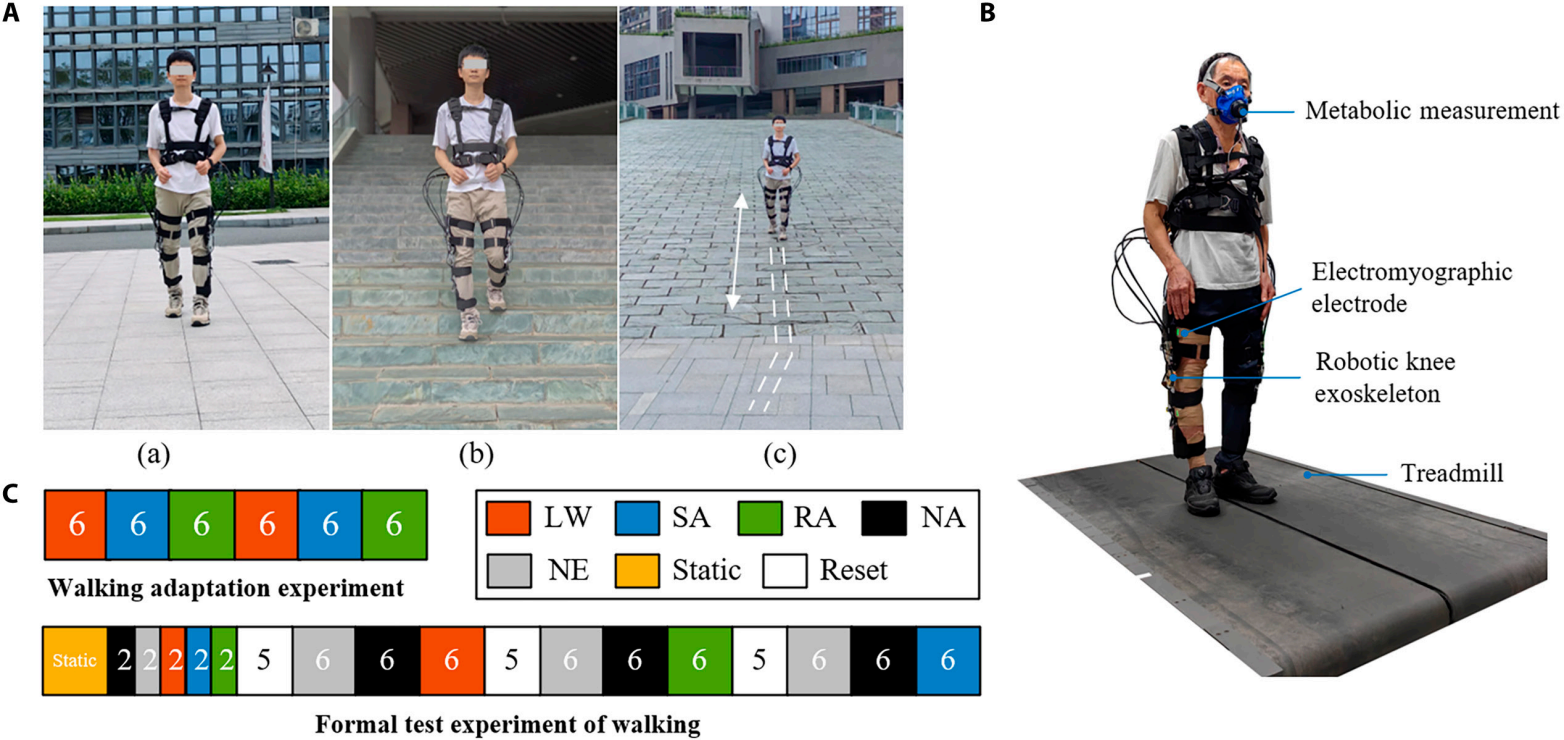
Real outdoor experiment settings and indoor experiment procedure design. (A) Level walking (a), stair (b), and ramp (c) scenarios. (B) Indoor measurement of energy loss. (C) Experimental procedure of measuring the energy loss.

The performance of the proposed LPR method was evaluated using real outdoor scenarios, including level ground, stairs, and ramps as presented in Fig. 6A. The stair was 16 cm in height and 30 cm in depth, and the ramp had a slope of 10°. For the stair scenario, participants were required to perform the following sequence of activities: (a) Standing → (b) LW → (c) SA → (d) LW → (e) SD → (f) LW → (g) standing. Similarly, participants were also to perform the following activities during the ramp scenario: (a) Standing → (b) LW → (c) RA → (d) LW → (e) RD → (f) LW → (g) standing.

#### Energy consumption experiment of exoskeleton

As in Fig. 6B, to evaluate the performance of the finite state time-sharing auxiliary control strategy for FKAE under 3 different motion modes (LW, SA, and RA), the metabolic cost and surface muscle signal changes of participants were measured and statistically analyzed under different motion conditions and control conditions (walking without the exoskeleton). By comparing the average metabolic cost and surface muscle signal data of participants walking with and without exoskeleton, the effectiveness of the proposed auxiliary strategy was verified, as well as the patterns of average metabolic cost and surface muscle signal changes under different motion modes with assistance of the exoskeleton. Oxygen consumption (${\mathrm{VO}}_{2}$) and carbon dioxide production (${\mathrm{VCO}}_{2}$) during walking were measured through indirect calorimetry using a wearable metabolic system—K5 (COSMED, Italy). Net metabolic power (W/kg) was computed by applying the modified Brockway equation [43] to the measured gas exchange data. To isolate task-specific energy expenditure, resting metabolic rate values were systematically deducted from. The robot designed in this paper provides auxiliary assistance for the wearer’s knee flexion during LW, RA, and SA, so the muscle groups related to knee walking (semitendinosus, biceps femoris, gastrocnemius, tibialis anterior, rectus femoris) [44,45] were recorded at 1,000 Hz for analysis with an electromyography measurement system (Trigno Avanti Sensor, DELSYS, USA), with noninvasive electromyography electrodes affixed to the muscle bellies according to European standards. Since there is a certain coupling relationship between the knee and ankle joint movements during walking, this paper also selects the tibialis anterior muscle data related to the ankle-foot clearance process [46,47]. Due to the significant individual differences in the sEMG signal even at the same muscle site, it was necessary to calibrate the target muscles of each participant before they participated in the experimental tasks, by performing specific actions to establish standardized baseline values for individual muscle sites. Raw EMG signals underwent digital processing with a fourth-order Butterworth band-pass filter (20 to 400 Hz cutoff frequencies), followed by full-wave rectification and phase-corrected low-pass filtering (6 Hz cutoff, second-order) to generate linear envelopes. Amplitude normalization was performed by scaling each muscle’s envelope to its peak activation magnitude observed during unloaded normal walking (UN) trials. Subsequent analysis involved computing root mean square (RMS) values (200 ms moving window) of the normalized envelopes, with cross-condition statistical comparisons implemented to quantify activation pattern divergences [48].

As presented in Fig. 6C, the experimental protocol included 2 phases: phase I, the walking adaption phase; phase II, the formal test experiment of walking under different motion modes. During the phase I adaption experiment, participants adapted to each exercise mode condition twice, each lasting 6 min. In the phase II formal experiment, human metabolic cost and sEMG signals were measured simultaneously. Before the formal test, the participants stood still to measure the baseline value of metabolic costs. All participants randomly selected a motion mode for warm-up and walked for 2 min in each mode. After a 5-min break, participants began the formal experiments, which included 5 conditions: 3 motion mode conditions wearing the exoskeleton with assistance (LW, RA, and SA), wearing the exoskeleton without assistance (NA), and walking without wearing the exoskeleton (NE, as the blank control group). Participants randomly selected a motion mode for the experiments, walking for 6 min in each mode and took a 5-min rest between each experimental condition. The order of experimental conditions was randomized to minimize learning effects. Data from the last 2 min for each condition were adopted for processing and statistical analysis.

#### Performance evaluation

1. Evaluation indicators of SSDD Actuation switching performance: The relationship was conducted between the switching time $T_{p}$ of SSDD actuation from one side to the other and the user’s gait period $T_{m}$ (m representing the motion mode) in different motion modes. It is further adopted to verify whether the switching performance of SSDD actuation satisfies the requirements of performance.

2. Performance indicators of the LPR method: Two indicators were adopted to evaluate the performance of the proposed LPR algorithm: success rate and detection delay. To provide a reasonably reliable assessment of recognition performance, the overall classification accuracy (RA) and single-mode classification accuracy (CA) for each locomotion mode for each participant were calculated via the following equation:

$$\left[\mathrm{RA}\;\mathrm{CA}\left(m\right)\right]=\left[\begin{array}{ll} \frac{100N_{r}}{N_{t}} & \frac{100N_{\mathrm{mr}}}{N_{m}} \end{array}\right]$$ (9)

where *m* represents the motion mode, $N_{r}$ represents the number of correct recognition results, $N_{t}$ represents the total number of recognition results, $N_{\mathrm{mr}}$ represents the number of successful recognition results in the motion mode m, and $N_{m}$ represents the total number of recognition results in the motion mode m.

To evaluate whether the motion transition was detected in time, the detection delay during the motion transition was calculated by the following equation:

$$\left[\begin{array}{ll} T_{t}\left(m\right) & P_{t}\left(m\right) \end{array}\right]=\left[\begin{array}{ll} t_{p}-t_{r} & \frac{100T_{t}\left(m\right)}{T_{c}\left(m\right)} \end{array}\right]$$ (10)

where $t_{p}$ represents the time when the transition is detected, $t_{r}$ represents the time when the actual transition is completed, and $T_{c}$ represents the average duration of one gait cycle of a specific motion mode within a stable state. $T_{t}$ represents the delay time, and $P_{t}$ represents the percentage of the delay time relative to the total time in one gait cycle.

3. Evaluation indicators for finite state time-sharing auxiliary control strategy: in order to verify the feasibility and effectiveness of the finite state time-sharing auxiliary control strategy in 3 different motion modes (LW, SA, and RA). Laboratory experiments were conducted with participants wearing the knee assistive exoskeleton and performing gait activities in different motion modes. The average metabolic cost and surface muscle signal of participants in different motion mode conditions and control conditions (compared with walking without wearing the exoskeleton) were measured and analyzed simultaneously. The ROC indicator was adopted to measure the percentage of average value changes in the average surface myoelectric signal (sEMG) of the participant’s muscle groups, which are collected after performing activities with wearing the exoskeleton with assistance (WA) and wearing the exoskeleton without assistance (NA), respectively.

$$\mathrm{ROC}=\left|\frac{V_{\mathrm{WA}}}{V_{\mathrm{NA}}}-1\right|\times 100\%$$ (11)

where $V_{\mathrm{WA}}$ represents the average sEMG value of the participant performing activities wearing the exoskeleton with assistance (WA), and $V_{\mathrm{NA}}$ represents the average sEMG value of the participant performing activities wearing the exoskeleton without assistance (NA).

### Results

#### SSDD actuation switching performance results

Table 4 presents the participants’ average gait cycle $T_{m}$ (s) and actuator switching time $T_{p}$ (s) in different motion modes. The switching duration of legs accounts from 12% to 20% of the total gait cycle in different motion modes. According to the results in Table 3, the value range of the average gait cycle $T_{m}$ of the participants under different motion modes is $T_{m}\in [1.14,\;1.28]\;$, and the value range of the driver switching time $T_{p}$ is $T_{p}\in \left[0.10,\;0.12\right]$. The value range of driver switching time $T_{p}$ satisfies the average switching time range of participants in different motion modes $\left(12\%∼20\%\right)\cdot T_{m}\in \left[0.14,\;0.26\right]$. Therefore, the switching performance of the driver satisfies the motion requirements of users wearing the exoskeleton. Furthermore, the elderly possesses a longer gait cycle than the young due to decreased muscle strength and flexibility and weakened ability of controlling the gait movement, thus reflected as longer support phase in the gait cycle and a shorter swing phase to stabilize the body with preventing falls [49]. Addressed with descriptions above, the switching performance of the driver satisfies the motion requirements of both young and elderly users.

**Table 4.** Mean gait cycle $T_{m}$ (s) for subjects in different movement modes and actuation switching time $T_{p}$ (s)

$T_{m}$	$T_{\mathrm{LW}}$	$T_{\mathrm{SA}}$	$T_{\mathrm{SD}}$	$T_{\mathrm{RA}}$	$T_{\mathrm{RD}}$
0.10−0.12	1.16	1.24	1.14	1.28	1.15

#### Locomotion mode recognition experimental results

**Fig. 7.**
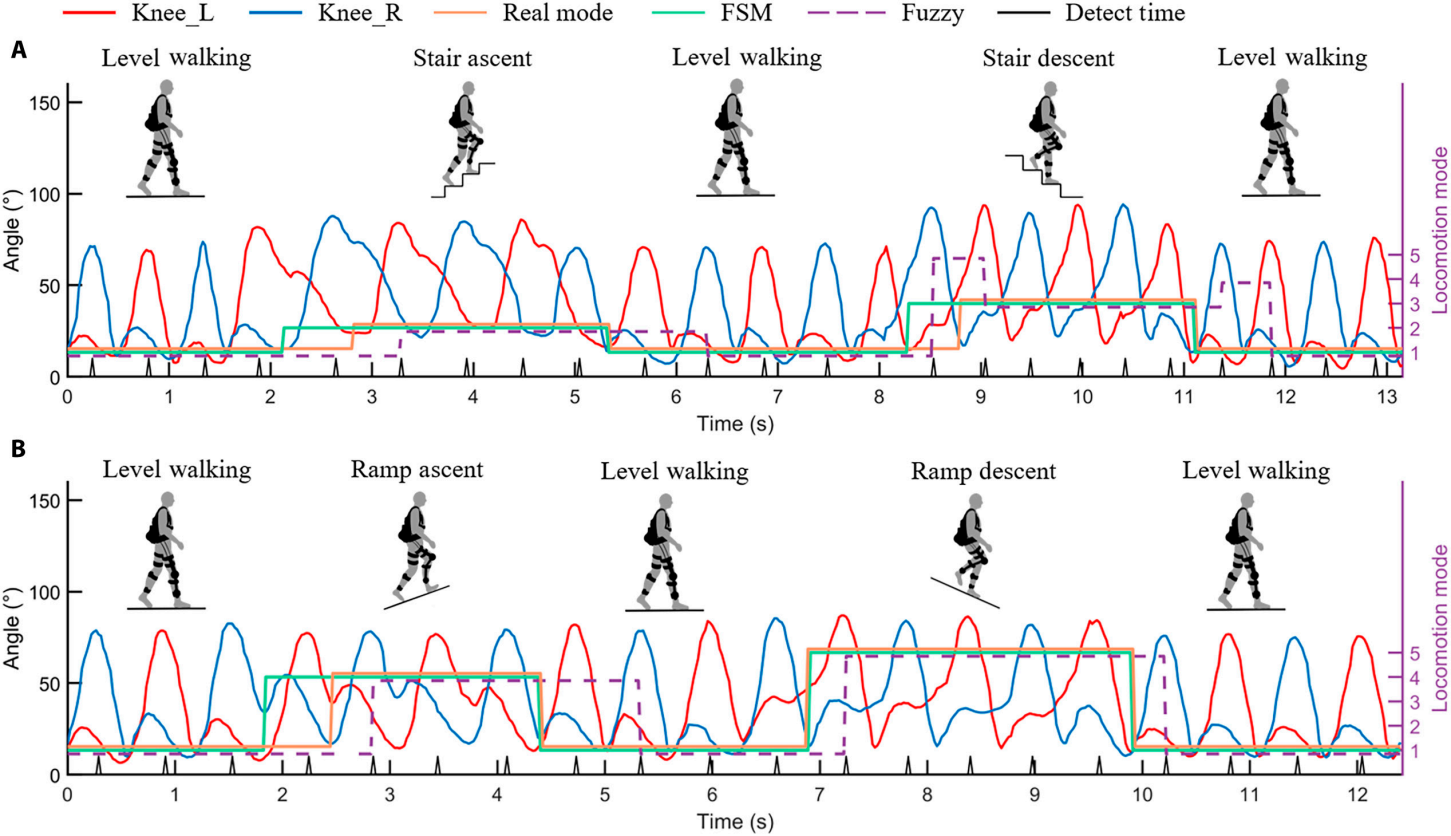
Cyclic locomotion pattern recognition results of dual recognition strategy based on fuzzy control and finite state machine algorithm. (A) Cyclic motion with mixture of level walking and stairs ascending/descending. (B) Cyclic motion with mixture of level walking and ramps ascending/descending.

Fig. 7 presents the cyclic LPR results of participants performing activities under scenarios of stairs and ramps based on the dual recognition strategy of fuzzy control and FSM algorithm. In the stair scenario, recognition algorithm with fuzzy control only experienced issues with misidentification motion modes and significant delays during motion transitions. However, integrating steady-state fuzzy control with FSM recognition algorithms substantially reduces delays and eliminates misidentification issues during motion mode transitions. The introduction of the FSM algorithm even recognizes motion mode switching of users in advance, as presented in Fig. 7A. Similarly, the combined steady-state fuzzy control with FSM recognition algorithms effectively reduces delays during motion mode transitions in the ramp scenarios, as presented in Fig. 7B.

**Fig. 8.**
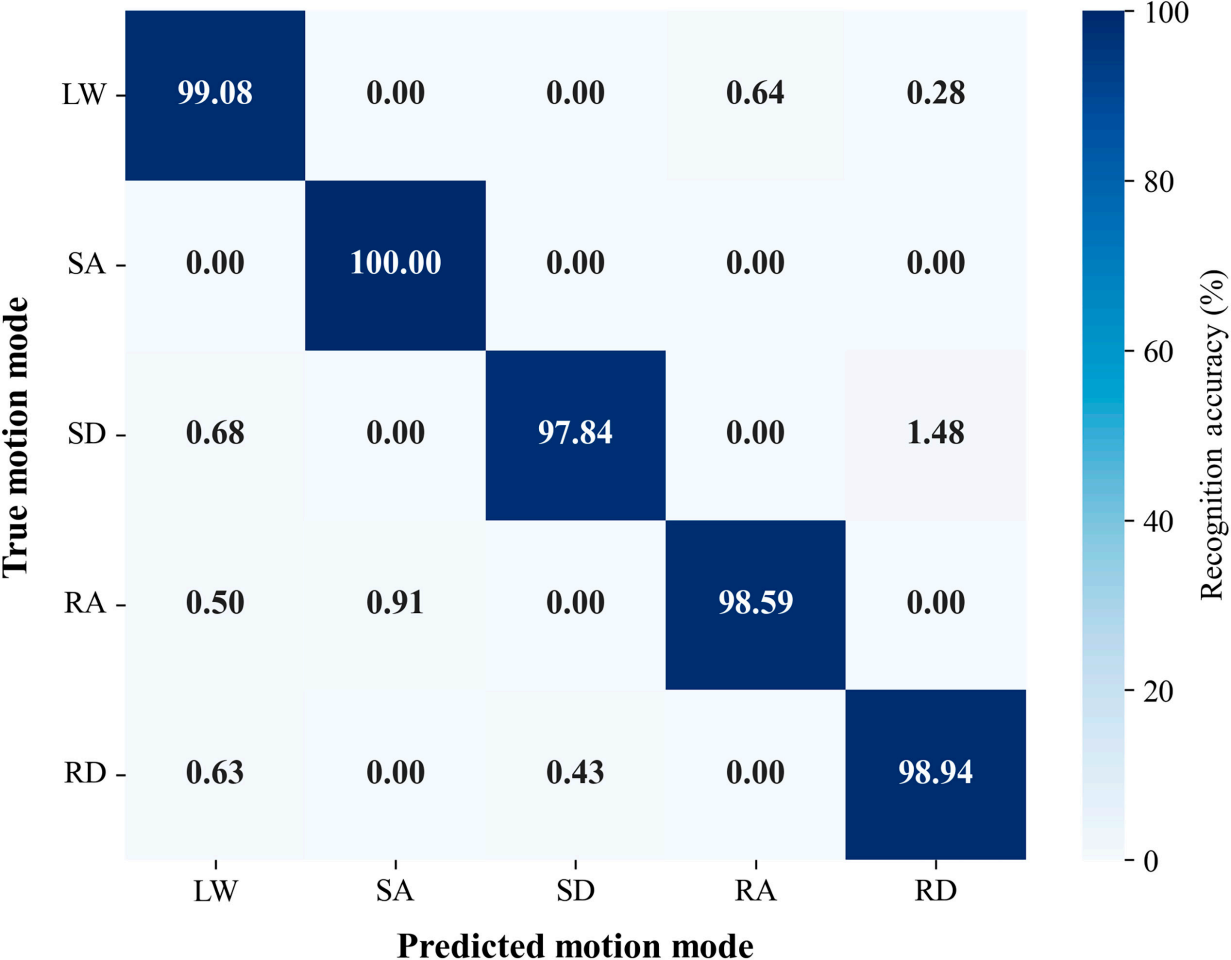
Confusion matrix of the proposed LPR strategy across 5 different locomotion modes.

The recognition accuracy of steady-state mode is defined as the ratio of the accurately recognized gait instances to the total number of gait instances, as presented in Table 5. During the steady-state motion mode recognition experiments, the motion trajectories (scenarios of level ground, stairs, and ramps) were fixed, and participants walked at a self-selected speed without any mode transitions. Under these conditions, all young participants achieved a 100% recognition accuracy in the LW condition. The elderly participant occasionally experienced gait misidentifications in the LW condition, resulting in a recognition accuracy slightly below 100%. All participants had a 100% recognition accuracy in the SA condition and an average accuracy rate of 97.84% in the SD condition. The average recognition accuracy for the RA and RD conditions reached 98.59% and 98.94%, respectively, with an overall average accuracy rate above 98% throughout the steady-state motion mode recognition experiments. Fig. 8 details the LPR framework’s classification performance and inter-subject variability via a confusion matrix. The algorithm achieves robust true-positive rates (exceeding 98% overall), with off-diagonal elements transparently revealing the distribution of minor misclassifications.

**Table 5.** Results of the LPR process

	CA (LW)		CA (SA)		CA (SD)		CA (RA)		CA (RD)		RA	
	Nos.	Accuracy /%	Nos.	Accuracy /%	Nos.	Accuracy /%	Nos.	Accuracy /%	Nos.	Accuracy /%	Nos.	Accuracy /%
#1	216	100	108	100	108	99.07	156	100	160	98.75	748	99.56
#2	208	100	108	100	108	100	156	98.78	164	100	744	99.77
#3	210	100	108	100	108	97.22	160	97.56	154	100	740	98.96
#4	220	97.22	108	100	108	95.37	164	98.78	166	98.70	766	98.01
#5	228	98.86	108	100	108	98.54	162	98.56	164	97.64	770	98.72
#6	224	98.42	108	100	108	96.86	164	97.88	170	98.56	774	98.34
All	1306	99.08	648	100	648	97.84	962	98.59	978	98.94	4542	98.89

Table 6 presents the average delay time $T_{t}$ (s) and the percentage of delay time $P_{t}$ (%) of 6 participants during the experiment of motion mode switching. The results in Table 6 present negative value of delay time in recognizing switching from LW to other motion modes, demonstrating that the motion pattern recognition algorithm recognizes the switching of motion mode in advance. The value of delay time of switching from other motion modes to LW mode is minor, and even reaching zero in many cases. It suggests that the recognition of changes in user motion modes by the algorithm was nearly simultaneous with the actual moment of change in user motion modes.

**Table 6.** Mean delay time *T*_*t*_ (s) and detection latency percentage *P*_*t*_ (%) in the real-time experiments for subjects

Locomotion Mode Transition	#1		#2		#3		#4		#5		#6		All	
	*T*_*t*_/s	*P*_*t*_/%	*T*_*t*_/s	*P*_*t*_/%	*T*_*t*_/s	*P*_*t*_/%	*T*_*t*_/s	*P*_*t*_/%	*T*_*t*_/s	*P*_*t*_/%	*T*_*t*_/s	*P*_*t*_/%	*T*_*t*_/s	*P*_*t*_ /%
LW → SA	−0.6	−46.3	−0.8	−59.6	−1.0	−71.3	−1.0	−72.1	−1.0	−62.5	−0.7	−75.2	−0.85	−64.5
SA → LW	0	0	0	0	0	0	0	0	0	0	0	0	0	0
LW → SD	−0.7	−60.71	−0.7	−63.4	−1.0	−92.0	−1.0	−86.6	−0.85	−75.67	−0.74	−77.65	−0.83	−76.0
SD → LW	0	0	0	0	0	0	0	0	0	0	0	0	0	0
LW → RA	−0.66	−57.89	−0.6	−54.4	−0.6	−52.3	−0.6	−53.5	−0.6	−54.4	−0.64	−56.2	−0.62	−54.8
RA → LW	0	0	0	0	0	0	−0.1	−6.1	−0.1	−4.6	−0.1	−6.2	−0.05	−2.8
LW → RD	−0.6	−58.49	−0.7	−61.3	−0.7	−64.2	−0.6	−59.4	−0.7	−61.3	−0.7	−63.2	−0.67	−61.3
RD → LW	−0.1	−11.4	0	0	0	0	−0.2	−15.1	−0.1	−6.6	−0.1	−10.2	−0.08	−5.32

### Experimental results of energy consumption

**Fig. 9.**
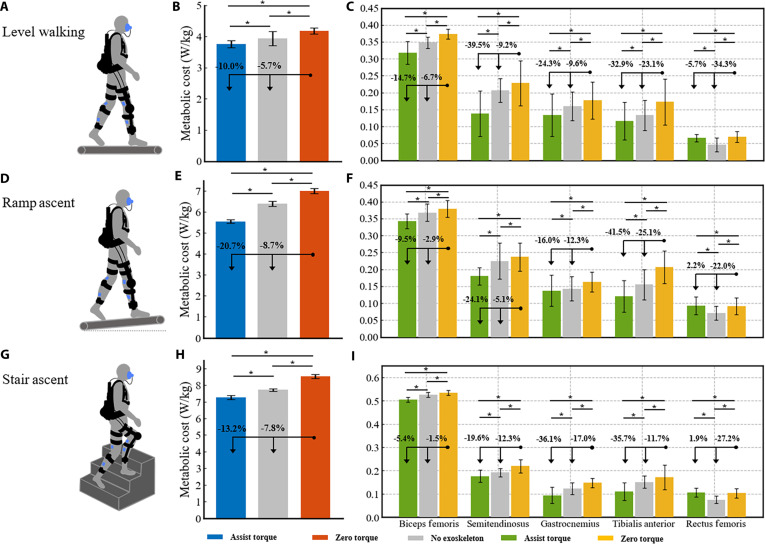
Effects of exoskeleton on the average metabolic cost and surface muscle signals of users while walking. (A, D, and G) Participant’s metabolic costs and surface muscle signals measured in scenarios of level walking, RA, and SA, respectively. The measurements include conditions that the user wears the exoskeleton with assistance (providing assistance), the user wears the exoskeleton without assistance (zero torque), and the user does not wear the exoskeleton (natural state). (B, E, and H) Average metabolic costs for all users under different motion modes, with arrows indicating the percentage reduction compared to zero-torque condition. (C, F, and I) Average changes in surface muscle signals during the experiments across different motion modes, with arrows indicating the percentage reduction compared to the zero-torque condition. *P<0.05.

The changes in average metabolic costs and average surface muscle signals were measured for 6 participants performing walking tasks in scenarios of LW, RA, and SA under 3 conditions: wearing exoskeletons with assistance, wearing exoskeletons without assistance (zero torque, NA), and not wearing exoskeletons (natural state, NE), as presented in Fig. 9. In scenarios of LW, the auxiliary torque significantly reduced the average metabolic cost by 0.22 W/kg ± 0.07 W/kg (4.3% ± 1.8%, P<0.05) compared with state of NE, and by 0.42 W/kg ± 0.09 W/kg (10.0% ± 2.1%, P<0.05) compared with NA, as presented in Fig. 9B. The auxiliary torque reduced the surface muscle signal by up to 30.3% compared with NE and 39.5% compared with NA, as presented in Fig. 9C. In scenarios of RA, the auxiliary torque significantly reduced the mean metabolic cost by 0.84 W/kg ± 0.03 W/kg (12.0% ± 0.5%, P<0.05) compared with NE, and by 1.45 W/kg ± 0.07 W/kg (20.7% ± 1.0%, P<0.05) compared with NA, as presented in Fig. 9E. The auxiliary torque reduced the surface muscle signal by up to 16.4% compared with NE and by up to 41.5% compared to zero torque, as presented in Fig. 9F. Additionally, in scenarios of SA, the auxiliary torque significantly reduced the metabolic cost by 0.45 W/kg ± 0.06 W/kg (5.4% ± 0.8%, P<0.05) compared with NE, and by 1.26 W/kg ± 0.05 W/kg (13.2% ± 6.5%, P<0.05) compared with zero torque, as presented in Fig. 9H. The auxiliary torque reduced the surface muscle signal by up to 24.0% compared with NE and up to 35.7% compared with zero torque, as presented in Fig. 9I.

**Fig. 10.**
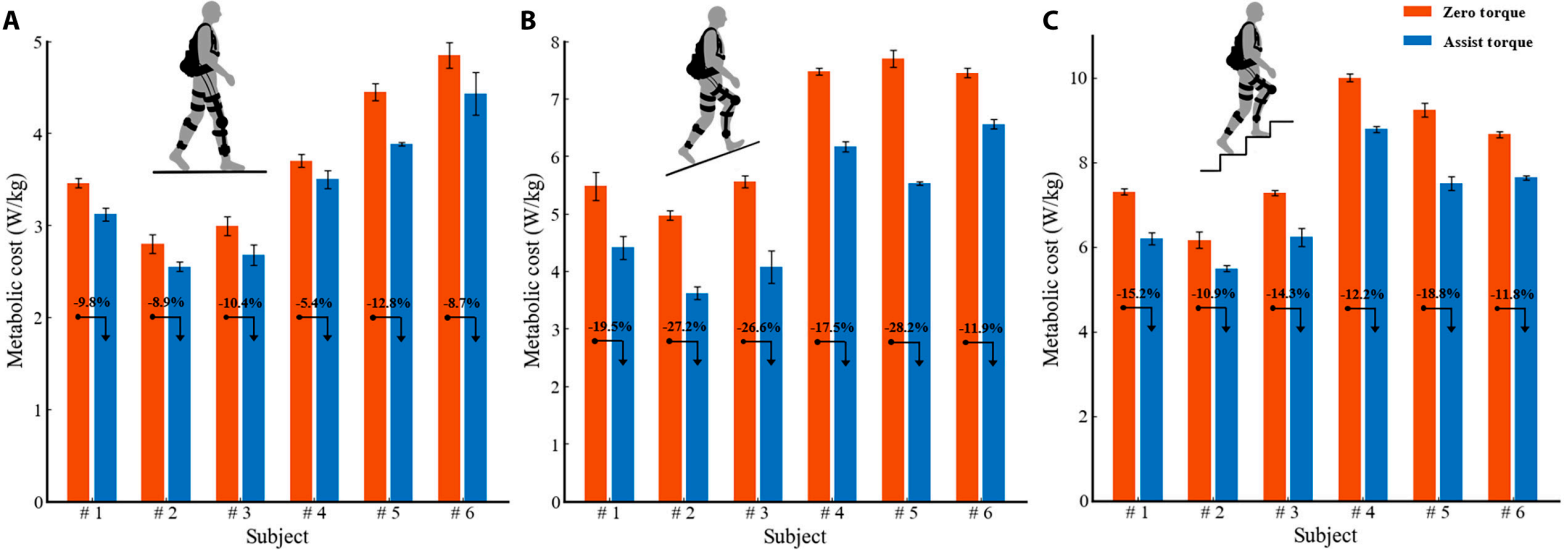
Changes of the average metabolic cost of participants under different motion modes. Scenes of level walking (A), ramp ascending (B), and stair ascending (C).

As presented in Fig. 10, wearing exoskeleton with assistance reduces the average metabolic cost of the user in different motion modes. In scenarios of LW, the average metabolic cost of participants 1, 2, 3, 4, 5, and 6 with exoskeleton assistance was reduced by 9.8%, 8.9%, 10.4%, 5.4%, 12.8%, and 8.7%, respectively, compared to zero-torque state. In scenarios of RA, the average metabolic cost of participants 1, 2, 3, 4, 5, and 6 with exoskeleton assistance was reduced by 19.5%, 27.2%, 26.6%, 17.5%, 28.2%, and 11.9%, respectively, compared to zero-torque state. In scenarios of SA, the average metabolic cost of participants 1, 2, 3, 4, 5, and 6 with exoskeleton assistance was reduced by 15.2%, 10.9%, 14.3%, 12.2%, 18.8%, and 11.8%, respectively, compared to zero torque. The results demonstrate the effectiveness of the exoskeleton-assisted strategy in reducing metabolic costs for both younger and older participants. Compared to the zero-torque state, the assistive strategy reduced the metabolic cost for participants 1 to 6 by 5.4% to 12.8% in LW, 11.9% to 28.2% in RA, and 10.9% to 18.8% in SA. These individual trends support the feasibility of the proposed assistive strategy in reducing metabolic effort across this specific cohort.

**Fig. 11.**
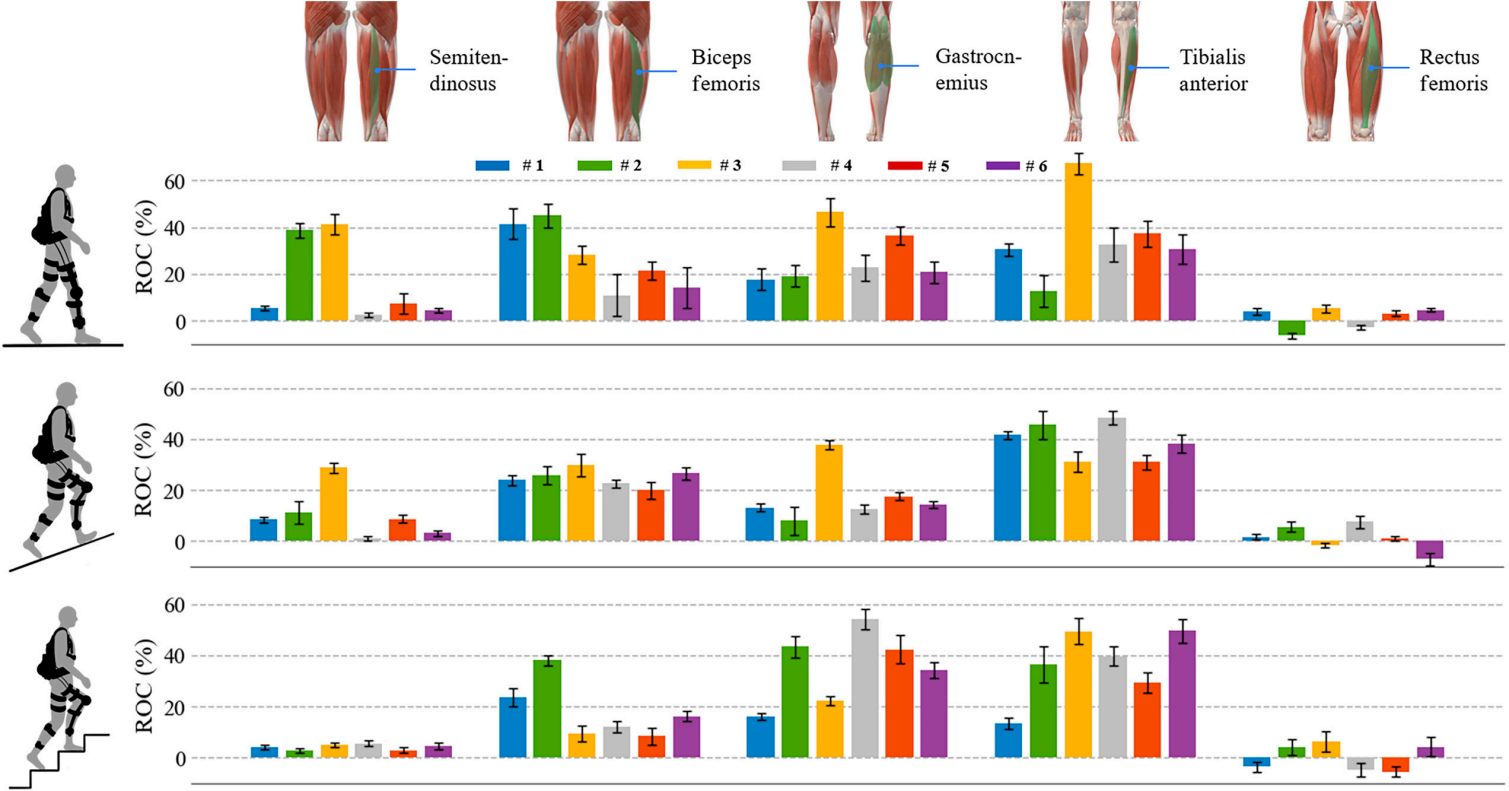
Changes in surface muscle signals (semitendinosus, biceps femoris, gastrocnemius, tibialis anterior, and rectus femoris) of 6 subjects under different movement modes (LW, RA, and SA). The rate of change (ROC) indicator was adopted to measure the percentage of average value changes in the average sEMG of the participant’s muscle groups, which are collected after performing activities with wearing the exoskeleton with assistance (WA) and wearing the exoskeleton without assistance (NA), respectively.

As demonstrated in Fig. 11, exoskeleton assistance reduces the user’s surface muscle signals under different motion modes. In scenarios of LW, the ROC of surface muscle signals of participant’s different muscle groups (semitendinosus, biceps femoris, gastrocnemius, and tibialis anterior) with exoskeleton assistance was reduced by 41.40%, 45.05%, 46.43%, and 67.23%, respectively, compared to a zero-torque state. In scenarios of RA, the ROC of muscle signals on the surface of different muscle groups was reduced by 28.75%, 29.90%, 37.67%, and 48.29%, respectively, with exoskeleton assistance compared with zero torque. In scenarios of SA, the ROC of surface muscle signals of different muscle groups was reduced by 5.66%, 38.06%, 43.36%, and 49.66%, respectively, with exoskeleton assistance compared with zero torque. The results demonstrate the effectiveness of the exoskeleton-assisted strategy in reducing surface muscle signals for both younger and older participants. Since the FKAE designed in this paper is mainly used to assist users in lifting their legs, it has basically no assisting effect on the extensor muscle group—the rectus femoris. These detailed muscle-level evaluations further corroborate the exploratory viability of the system.

## Discussion

### Characteristics of the SSDD actuation

During daily walking, the human knee joints do not require continuous assistive torque from exoskeletons. Biomechanical analysis indicates that torque assistance is primarily needed during the mid-swing phase flexion, with a 50% gait cycle phase difference between the left and right lower limbs. Based on this physiological characteristic, a novel “single-source dual-drive” portable FKAE is designed in this paper. Firstly, the exoskeleton only needs a single drive motor to provide time-shared assistance to the left and right knee joints, reducing the number of high-power drive motors and reducing the weight of the entire system. Additionally, elastic elements are incorporated between the drive motor’s output and the load to alleviate the rigid shock caused by sudden changes in the state of the wire rope or accidental falls of the user. Furthermore, the FKAE utilizes a Bowden cable system between the DCCs and the knee joint actuators to provide assistance, positioning the drive source away from the joints at the body’s center of gravity. This configuration reduces the burden on the wearer and improves the overall portability of the exoskeleton. Experimental results in Table 4 demonstrate that the value range of driver switching time $T_{p}\in \left[0.10,\;0.12\right]$ meets the required average switching time range of subjects in different motion modes $\left(12\%∼20\%\right)\cdot T_{m}\in \left[0.14,\;0.26\right]$, thus ensuring that the actuator’s performance satisfies the needs of users in various activities. It is worth noting that the older people usually exhibit longer gait cycles than do younger people, which typically range from 1.5 to 2 s. The elderly tends to perform slower paces, shorter stride length, and longer gait cycles due to the decrease in muscle strength, balance, and flexibility [41]. Hence, the required performance of actuator when performing assistance is lower than that for younger users. Based on the descriptions above, the actuator’s performance is also capable of meeting the motion needs of elderly users in daily activities.

### Advantages of the proposed locomotion mode recognition method

1. Recognition accuracy rate during steady state: The real-world outdoor LPR experiments have verified the feasibility and effectiveness of the recognition algorithm based on fuzzy control for the accurate recognition of users’ daily motion patterns (LW, SA, SD, RA, and RD) under steady-state conditions. Experimental results in Table 5 demonstrates that the average accuracy of all participants in the entire steady-state motion pattern recognition experiment reaches 98.89%. The maximum accuracy of young participants (#1, #2, #3) in the entire steady-state LPR experiment is 99.77%, while the maximum accuracy of older participants (#4, #5, #6) also achieved 98.72% accuracy. The experimental results demonstrate the effectiveness of the steady-state LPR algorithm based on fuzzy control, which can not only accurately and precisely identify the motion patterns of young participants under steady-state conditions but also apply to elderly participants. The recognition performance of this method is better than that of the effective recognition methods proposed for active prosthetic devices in Refs. [17,50,51] and also better than the reported average accuracy of 97.7% in Ref. [52]. Although the method of integrating mechanical sensing with sEMG signal can further make the recognition accuracy close to 99% [53,54], the changes of skin conditions during movement can cause the degradation of sEMG interface over time, thereby reducing its accuracy and reliability in long-term use. For the method based only on mechanical induction, recognition accuracy of the proposed method is also higher than that of the method based on threshold FSM [55]. Kinematic differences between subjects do affect LPR performance, and the threshold is sensitive to small changes in the recorded kinematics. A fuzzy control algorithm similar to ours was proposed in Ref. [37], but our LPR presents higher accuracy. The average accuracy of 97.7% in Ref. [37] performed 1.38% lower than the global average accuracy of 99.08% by our method.

2. Detection latency during toggle state: The outdoor motion mode transition experiments inducted 2 main results. One was the transition from LW to other motion modes (SA, SD, RA, and RD), where the detection delays were negative and the percentage delay was less than −50%. Experimental results in Table 6 indicate that the LPR algorithm could anticipate a user’s change in motion mode by half a gait cycle. The other result was transitions from other motion modes back to LW, where the detection delays were zero or near zero, indicating that the LPR algorithm could detect changes in the user’s motion mode almost simultaneously with the actual change. The reason why the change of the user’s movement mode can be recognized half a gait cycle in advance when the user switches from the LW mode to other modes is that, when any leg of the user switches from the LW mode to other modes, the leg has already performed joint kinematic characteristics in other modes, which are quite different from the LW mode. Therefore, waiting for the other leg to complete the switching action is not necessary. However, when transitioning from other modes back to LW, both legs must complete their transitions before a change in motion mode can be detected. This is because when a leg switches from another mode to LW, it is essentially still completing the previous motion mode. The change is more gradual, and the kinematic characteristics are not significantly different from the previous step, so it is necessary to wait for the other leg to complete its transition as well. Moreover, the method proposed in this paper is superior to the existing literature [9] (unilateral exoskeleton) and [26] (bilateral exoskeleton), in which there is a half-step or one-step detection delay during mode transition. Seamless exoskeleton assistance typically requires state transition detection within a strict 100- to 150-ms window to prevent human–robot conflict. As shown in Table 7, the proposed Fuzzy-FSM framework far exceeds this baseline. It demonstrates robust predictive capabilities, yielding negative delay times (e.g., averaging −0.85 s for LW to SA transitions) that effectively anticipate terrain changes before the physical gait transition. Meanwhile, transitions returning to LW exhibit near-zero latency. By combining predictive anticipation with instantaneous detection, the strategy secures an ample temporal buffer for mechanical actuation, ensuring smooth and synchronized torque delivery.

**Table 7.** Results of the comparison with alternative LPR strategy process

LPR Strategy	CA (LW)	CA (SA)	CA (SD)	CA (RA)	CA (RD)
Threshold-based FSM	98.85	100	96.21	95.72	96.75
LDA	96.52	100	93.43	94.49	94.53
SVM	95.10	100	96.66	95.32	97.42
Fuzzy + FSM	99.08	100	97.84	98.59	98.94

Furthermore, an offline benchmark comparison was conducted using the existing experimental dataset to explore the relative advantages of the proposed Fuzzy-FSM framework (Table 7). The method was compared with conventional baseline algorithms, including a threshold-based FSM, LDA, and support vector machine (SVM) classifier. Preliminary results demonstrate that the Fuzzy-FSM framework achieves higher classification accuracy. Although learning-based models require extensive, task-specific datasets to optimize their performance, the Fuzzy-FSM approach natively offers reduced transition latency and smoother state switching during continuous walking. These characteristics render it particularly well-suited for real-time embedded deployment in FKAEs.

### Exoskeleton assistance based on finite state time-sharing assistance control strategy

In this paper, a finite state time-sharing auxiliary control strategy is proposed to control a power-assisted knee exoskeleton that provides external torque support to the user’s legs. The control strategy automatically adjusts the exoskeleton assistance to adapt to different users and motion modes. Furthermore, the effects of the auxiliary torque generated by the control strategy under the 3 motion modes (LW, SA, and RA) on the average metabolic cost and sEMG signal of different participants were also tested, as demonstrated in Fig. 9. Specifically, compared with no exoskeleton or zero torque, our method significantly reduces the average metabolic cost and the average sEMG signal for users in the scenarios of LW, RA, and SA. Fig. 10 presents the experimental results of changes in users’ metabolic cost under different motion modes, indicating that exoskeleton assistance can reduce the average metabolic cost in various motion settings. This assistance performs substantial effectiveness not only for young participants but also for the elderly ones. For elderly participants, the maximum metabolic cost was reduced by 12.8%, 28.2%, and 18.8%, respectively, in scenarios of LW, RA, and SA with exoskeleton assistance compared with zero-torque state. Moreover, the metabolic cost reduction achieved by the proposed method in this paper surpasses that reported in the existing literature [47] for various controllers (averaged reduction: 3.1% for biological torque, 2.9% for impedance controller, and 3.1% for proportional myoelectric controller). Fig. 11 presents the experimental results of changes in surface muscle signals under different motion modes, indicating that exoskeleton assistance can reduce the surface muscle signals of users in various activities. This reduction is substantial not only for young participants but also for the elderly. For elderly participants, the ROC in sEMG signals with exoskeleton assistance decreased by up to 37.23%, 48.29%, and 54.14%, respectively, during LW, RA, and SA compared with zero-torque state. The proposed method achieves greater sEMG reduction compared to Ref. [46], which reported average reductions of −16.0% for the vastus lateralis and −15.1% for the vastus medialis using a bilateral robotic knee exoskeleton, and Ref. [47], which reduced sEMG peaks of the rectus femoris by 34.8% and 39.5% during kneeling down and standing up motions, respectively. As shown in Fig. 11, when assisting the knee joint, the EMG intensity of the tibialis anterior muscle related to the ankle joint is also reduced. The results show that changing the dynamics of a lower limb joint can affect the dynamics of the non-assisted joint. Therefore, the knee exoskeleton can not only improve the walking or muscle efficiency of the knee joint but also improve the walking efficiency of the ankle joint or reduce the intensity of exercise, consistent with previous studies [46,47].

### Limitations and future works

Although the methods presented in this paper have achieved satisfactory results, there are still some limitations that need to be addressed. Firstly, the designed FKAE exoskeleton presents a design disadvantage compared to conventional knee exoskeleton systems [46,47,56] that utilize dual-drive motors to provide torque assistance throughout the entire gait cycle. Specifically, the FKAE system cannot simultaneously actuate bilateral exoskeleton joints, which fundamentally restricts its functional capabilities. This design constraint not only precludes the exoskeleton from providing assistance during tasks requiring symmetrical torque generation across both knees (e.g., jumping maneuvers) and cannot provide extension-assistive torque to the knee joints but also limits its ability to deliver continuous assistance through the complete stance phase of gait. Secondly, although the current LPR framework—relying primarily on 2 kinematic features ($\Delta \theta_{1}$ and $\Delta \theta_{2}$)—demonstrated high accuracy during this preliminary exploration in structured environments, its robustness under complex, real-world conditions warrants further consideration. Specifically, the system’s performance may be challenged by variations in walking speed, irregular terrains, turning motions, and nonperiodic movements. To address these limitations, future explorations will focus on enhancing the robustness of the LPR system by integrating supplementary sensor modalities (e.g., foot pressure insoles or sEMG) and developing adaptive algorithms capable of accommodating unconstrained, asymmetric, and nonperiodic locomotion in real-world scenarios. Thirdly, during the transition between motion modes, if a user’s leg falls on the boundary between 2 modes, the change in joint kinematics might not be distinct, leading to potential errors in recognizing the transition between motion modes. In the future, we will further optimize solutions for resolving issues during the transition process. Fourthly, the proposed finite state control strategy utilizes a predefined sinusoidal trajectory for assistive torque. While such fixed profiles guarantee the stability, safety, and simplicity essential for preliminary hardware exploration, they restrict the system’s ability to adapt to individual differences in gait, muscle strength, and metabolic cost. This uniform approach inherently limits the maximum assistive benefit. To address this, our future explorations will focus on integrating adaptive control and human-in-the-loop (HIL) optimization. By enabling real-time, subject-specific torque modulation based on physiological feedback (e.g., sEMG), these strategies will advance the FKAE from providing generalized baseline support to functioning as a highly personalized wearable robotic system. Finally, this study included 6 subjects (3 young and 3 elderly) in the feasibility and effectiveness exploration experiments for the FKAE device. This lack of statistical significance was observed, both in terms of individual differences and in the unspecified replicates performed on a single subject. In the future, we will increase the number of elderly subjects to more objectively demonstrate the performance of the FKAE device.

## Conclusion

A novel “single-source dual-drive” portable FKAE for daily activities is designed in this paper. Firstly, the exoskeleton proposes the SSDD solution, which utilizes a clutch to perform assistance to left and right knee joints with a single motor. Meanwhile, the exoskeleton incorporates cable-driven and spring series, improving the interaction comfort, preventing injuries from sudden rigid torque during daily activities, and providing structural safety. Secondly, a dual detection strategy integrating FSM and fuzzy control for cyclic motion mode recognition is proposed. This method accurately identifies common daily motion scenarios such as LW, SA, SD, RA, and RD. Kinematic characteristics captured by IMUs work as recognition feature parameters. The integration of fuzzy control facilitates the recognition of steady-state motion modes during dynamic walking, while the introduction of FSM improves the accuracy of motion mode transitions and reduces detection delays. Finally, a finite state time-sharing assistive torque control strategy based on the identified motion patterns is proposed to provide external torque support to the users legs through the powered knee exoskeleton. Compared to conditions without wearing the exoskeleton and to zero-torque state, this method can reduce the average metabolic cost and sEMG signals for users during experiments involving LW, RA, and SA. The structural designs and assistance features proposed in this paper not only are limited to lower limb knee exoskeletons but also may be applicable to the motion control of hip and ankle exoskeletons, upper limb rehabilitation exoskeletons, and prosthetics.

## Data Availability

Data to support the findings of this study are available from the first author upon request.
